# Integration features of intact latent HIV-1 in CD4^+^ T cell clones contribute to viral persistence

**DOI:** 10.1084/jem.20211427

**Published:** 2021-10-12

**Authors:** Amy S. Huang, Victor Ramos, Thiago Y. Oliveira, Christian Gaebler, Mila Jankovic, Michel C. Nussenzweig, Lillian B. Cohn

**Affiliations:** 1 Laboratory of Molecular Immunology, The Rockefeller University, New York, NY; 2 Howard Hughes Medical Institute, Chevy Chase, MD; 3 Fred Hutchinson Cancer Research Center, Seattle, WA

## Abstract

Latent intact HIV-1 proviruses persist in a small subset of long-lived CD4^+^ T cells that can undergo clonal expansion in vivo. Expanded clones of CD4^+^ T cells dominate latent reservoirs in individuals on long-term antiretroviral therapy (ART) and represent a major barrier to HIV-1 cure. To determine how integration landscape might contribute to latency, we analyzed integration sites of near full length HIV-1 genomes from individuals on long-term ART, focusing on individuals whose reservoirs are highly clonal. We find that intact proviruses in expanded CD4^+^ T cell clones are preferentially integrated within Krüppel-associated box (KRAB) domain–containing zinc finger (ZNF) genes. ZNF genes are associated with heterochromatin in memory CD4^+^ T cells; nevertheless, they are expressed in these cells under steady-state conditions. In contrast to genes carrying unique integrations, ZNF genes carrying clonal intact integrations are down-regulated upon cellular activation. Together, the data suggest selected genomic sites, including ZNF genes, can be especially permissive for maintaining HIV-1 latency during memory CD4^+^ T cell expansion.

## Teaser

This study combines HIV-1 sequence analysis and integration site discovery to show a preponderance of integrations into ZNF genes in expanded latent clones. ZNF genes, unlike other integration targets, are downregulated upon T cell activation, which may facilitate latency maintenance.

## Introduction

Antiretroviral therapy (ART) is highly effective in suppressing HIV-1 infection. Nevertheless, transcriptionally silent integrated proviruses persist in CD4^+^ T cells and can be reactivated upon treatment interruption or when stimulated to divide in vitro (Chun et al., 1997; Finzi et al., 1999; Wong et al., 1997). The latent compartment has an estimated half-life of 4–18 yr (Bachmann et al., 2019; Crooks et al., 2015; Peluso et al., 2020; Siliciano et al., 2003) and represents the primary barrier to HIV-1 cure.

Several different explanations have been considered for the long half-life of the latent reservoir, including infected cell proliferation. However, this idea is counterintuitive because stimuli that induce extensive CD4^+^ T cell division also activate HIV-1 gene expression, which in turn inhibits cell division and leads to cell death (Rambaut et al., 2004; Siliciano and Greene, 2011). Nevertheless, several independent studies estimate that 50–60% of all latent cells in chronically infected individuals are found in large, expanded clones and that the proportion of latent cells in expanded clones increases over time (Antar et al., 2020; Bui et al., 2017; Cohn et al., 2015; Hosmane et al., 2017; Lee et al., 2017; Lorenzi et al., 2016). The CD4^+^ T cells that comprise a given clone share the same HIV-1 proviral integration site and express a unique T cell receptor (Cohn et al., 2018; Simonetti et al., 2021). Notably, a significant fraction of these clonal T cells express antigen receptors that recognize viruses that cause chronic or recurrent infections, suggesting that expansion of latent clones is driven by antigen exposure (Mendoza et al., 2020; Simonetti et al., 2021).

The factors that determine whether an integrated provirus adopts a latent phenotype and its subsequent potential to be reactivated are not well understood. When considering all integrated proviruses, including the majority that are defective, integrations into *BACH2*, *MKL2*, and *STAT5B* are enriched (Bedwell et al., 2021; Cohn et al., 2015; Einkauf et al., 2019; Maldarelli et al., 2014; Wagner et al., 2014). However, whether these genes contribute to integration, selection, or latency remains unknown.

In contrast to defective proviruses, the integration landscape of intact proviruses has been more difficult to document in part because the cells that carry latent viruses represent a very minor fraction of all CD4^+^ T cells (Bruner et al., 2016; Ho et al., 2013). However, recent work has demonstrated that in chronically infected individuals on long-term ART, intact proviruses with the potential to be reactivated are enriched in nongenic regions, in an opposite transcriptional orientation to host genes (Einkauf et al., 2019). In contrast, integrated proviruses in individuals that control infection in the absence of ART, elite controllers (ECs), were enriched in regions of centromeric satellite DNA and in Krüppel-associated box (KRAB) domain–containing zinc finger (ZNF) genes (Jiang et al., 2020). The ECs also showed high-level enrichment of large clonal integrations (Boritz et al., 2016; Jiang et al., 2020; Veenhuis et al., 2018; Woldemeskel et al., 2020). Whether the distinct integration profile found in ECs represents a correlate of control or simply a feature that is especially permissive for clonal expansion has not been determined.

Here, we report on the analysis of intact proviral integration in expanded clones of latent cells in chronically infected individuals on long-term ART and how integration characteristics of this population compare to integration characteristics observed in ECs.

## Results

To characterize the integration features of latently infected CD4^+^ T cells, we used a modified matched integration site and proviral sequencing (MIP-seq) protocol (Einkauf et al., 2019; Halvas et al., 2020; Patro et al., 2019). The analysis focused on six individuals whose intact and replication competent proviruses had been extensively characterized through direct sequencing (quadruplex quantitative PCR [Q4PCR]; Gaebler et al., 2021; Gaebler et al., 2019) and viral outgrowth (quantitative and qualitative viral outgrowth assay; Fig. 2; Cohn et al., 2018; Lorenzi et al., 2016; Mendoza et al., 2018). The selected individuals were chronically infected and had been suppressed on ART for 4–21 yr (median, 13 yr; Table S1). As expected, the latent reservoirs in these volunteers were dominated by large-expanded clones of CD4^+^ T cells (Cohn et al., 2018; Gaebler et al., 2019; Mendoza et al., 2018).

**Figure 1.**
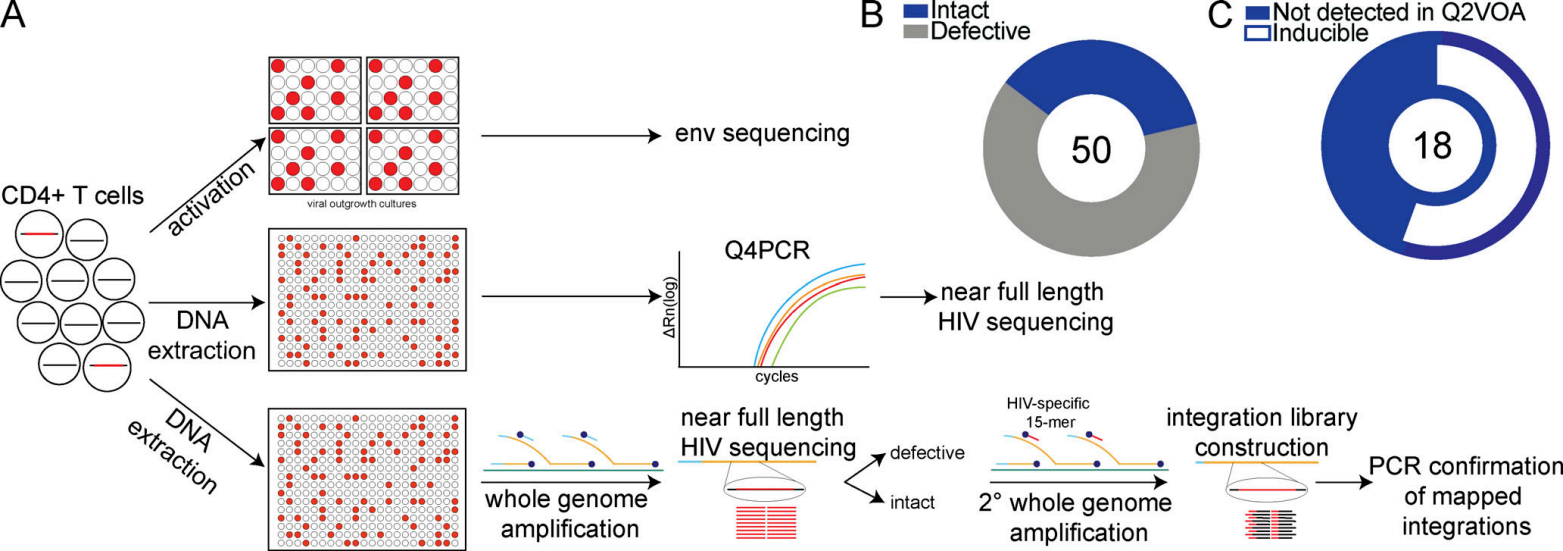
**Integrative analysis of latent reservoirs in ART-treated participants. (A)** Experimental design. *Env *sequences from inducible proviruses are obtained by limiting dilution viral outgrowth cultures (Lorenzi et al., 2016), and NFL sequences are obtained by Q4PCR (Gaebler et al., 2019). Paired NFL sequencing and integration site mapping is performed by modified MIP-seq (Einkauf et al., 2019). **(B)** Pie chart summarizes the total number of intact and defective proviral sequences mapped, center. Intact proviruses in blue, and defective in gray. **(C)** Pie chart shows the proportion of intact proviruses that are inducible in vitro in white. Intact proviruses not detected by quantitative and qualitative viral outgrowth assay (Q2VOA) represented in blue. The number in center indicates the total number of intact proviral integrations mapped.

To analyze HIV-1 sequences and determine their corresponding site of chromosomal integration, genomic DNA from CD4^+^ T cells was diluted to single proviral genome level and subjected to whole genome amplification (WGA; Fig. 1 A). *Env* and near full length (NFL) HIV-1 sequencing was performed on the WGA products. Viral sequence was compared with previously identified intact viruses (Gaebler et al., 2019; Mendoza et al., 2020), and proviruses were selected for integration site analysis by ligation-mediated PCR based on whether they were intact or defective. Through this approach, we focused the analysis on intact proviruses and proviral clones. Mapped integration sites were subsequently verified by integration site-specific PCR of WGA products. We mapped 50 unique integrations, 32 from defective proviruses and 18 from intact proviruses, 55.5% of which were inducible in outgrowth cultures (Fig. 1, B and C; and Table S2; Mendoza et al., 2018). Integration sites for identical sequences were counted as a single integration to avoid bias due to clonal expansion. Herein, we define unique or nonclonal proviruses as sequences detected only once in ex vivo assays while acknowledging the caveat that they may be members of small clones.

**Figure 2.**
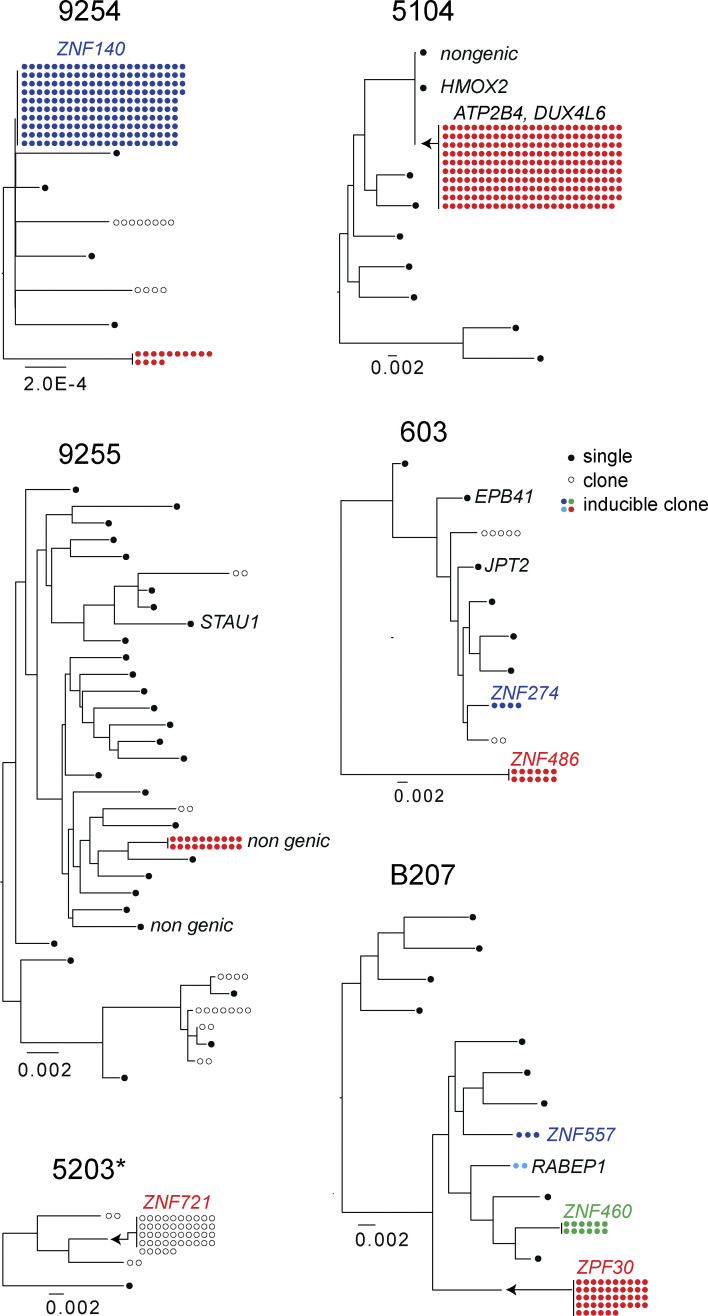
**Maximum likelihood phylogenetic trees of intact NFL sequences obtained by Q4PCR and MIP-seq (****Einkauf et al., 2019****; ****Lorenzi et al., 2016****).** Filled circles represent inducible clones, and the number of circles is proportional to the size of the clone. Outlined circles represent NFL HIV-1 clones detected by PCR, but not by outgrowth assays. Black circles represent unique proviruses detected only once. Integration sites indicated by adjacent text. *Viral outgrowth assay not performed.

Phylogenetic analysis was performed to compare the paired intact NFL proviral sequences obtained through MIP-seq to NFL viral genomes obtained by Q4PCR (Gaebler et al., 2019; Mendoza et al., 2020). Intact proviral sequences matching Q4PCR sequences were found in each of the six participants, confirming the absence of selection bias during WGA. Importantly, we captured members of the dominant clones from the reservoir of all six participants (Fig. 2). Across all individuals, the dominant clone corresponds to *env* sequences identified through outgrowth assays (Cohn et al., 2018; Laskey et al., 2016; Mendoza et al., 2018). Since *env* identity is a robust predictor of proviral clonality (Laskey et al., 2016), our analysis indicates that these large clones can produce infectious HIV-1.

**Figure 3.**
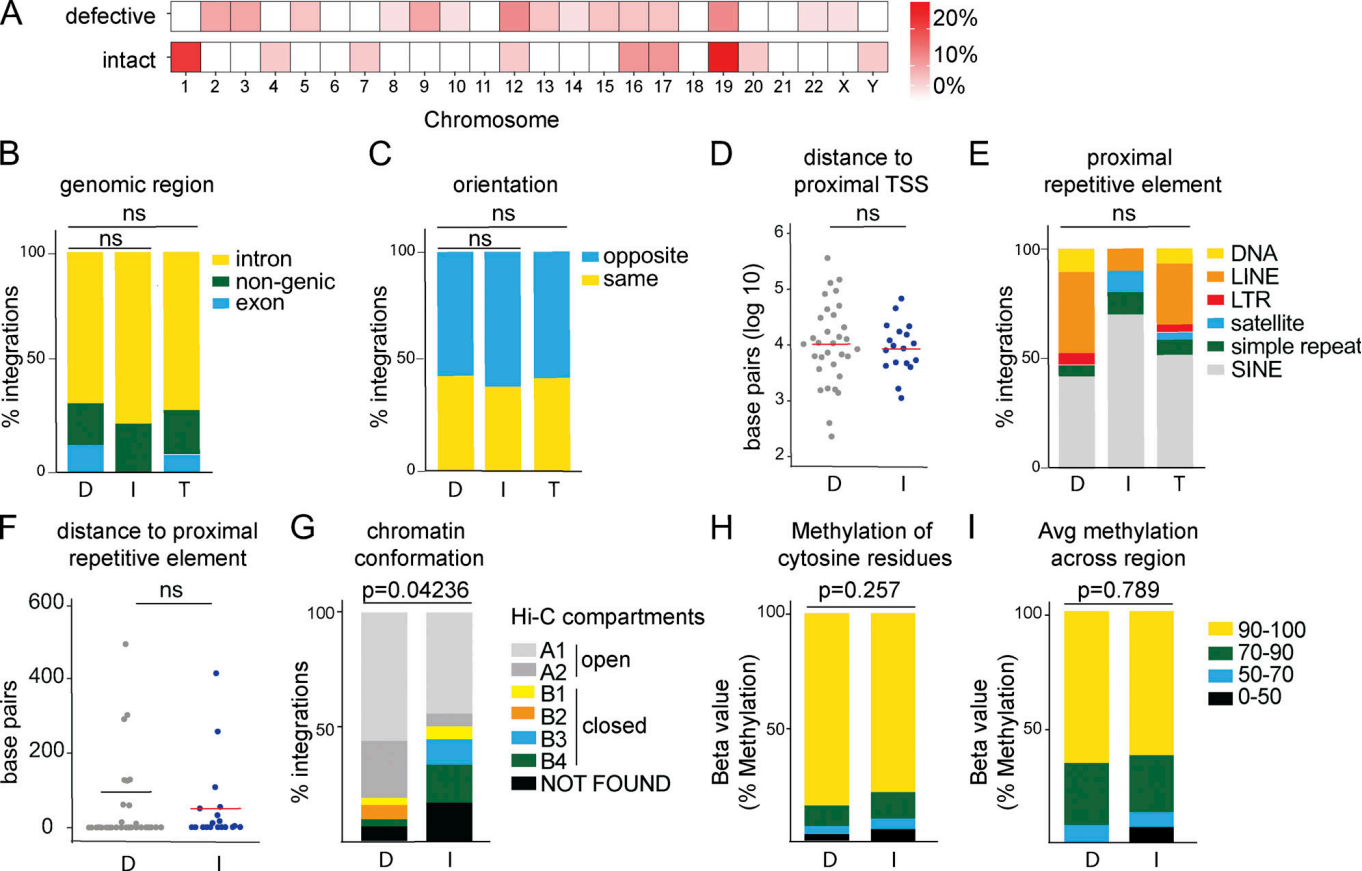
**Genomic features of defective (D), intact (I), and total (T) integration sites in ART-treated individuals. (A)** Upper heat map depicts proportion of HIV-1 defective integration sites in each chromosome; bottom heat map depicts proportion of HIV-1 intact integration sites in each chromosome (see Table S2). **(B)** Proportion of integrations in introns, exons, or nongenic regions. **(C)** Proportion of proviruses integrated in opposite or the same direction as host gene transcription. **(D)** Distance, in base pairs, of integration from nearest TSS. **(E)** Proportion of integrations in different repetitive elements classified by University of California, Santa Cruz RepeatMasker (Jurka, 2000). **(F)** Distance, in base pairs, of integrations from nearest repetitive element. **(G)** Proportion of intact and defective proviral sequences mapped within chromatin structural compartments A and B and their respective sub-compartments as determined by Hi-C sequencing data (see Table S2; Rao et al., 2014). P value refers to proportion of integrations in compartment A as determined by two-proportion Z-test. **(H)** Methylation 1,000 bp upstream of HIV-1 proviral promoter integration site in CD4^+^ T cells (Komaki et al., 2018). Proportion of intact and defective proviral integrations with average number of cytosine residues with indicated levels of methylation. **(I)** Proportion of intact and defective proviral integrations with indicated methylation levels for all residues. P values determined by two-tailed Fisher’s exact test. Avg, average; LINE, long interspersed nuclear element; SINE, short interspersed nuclear element.

**Figure S1.**
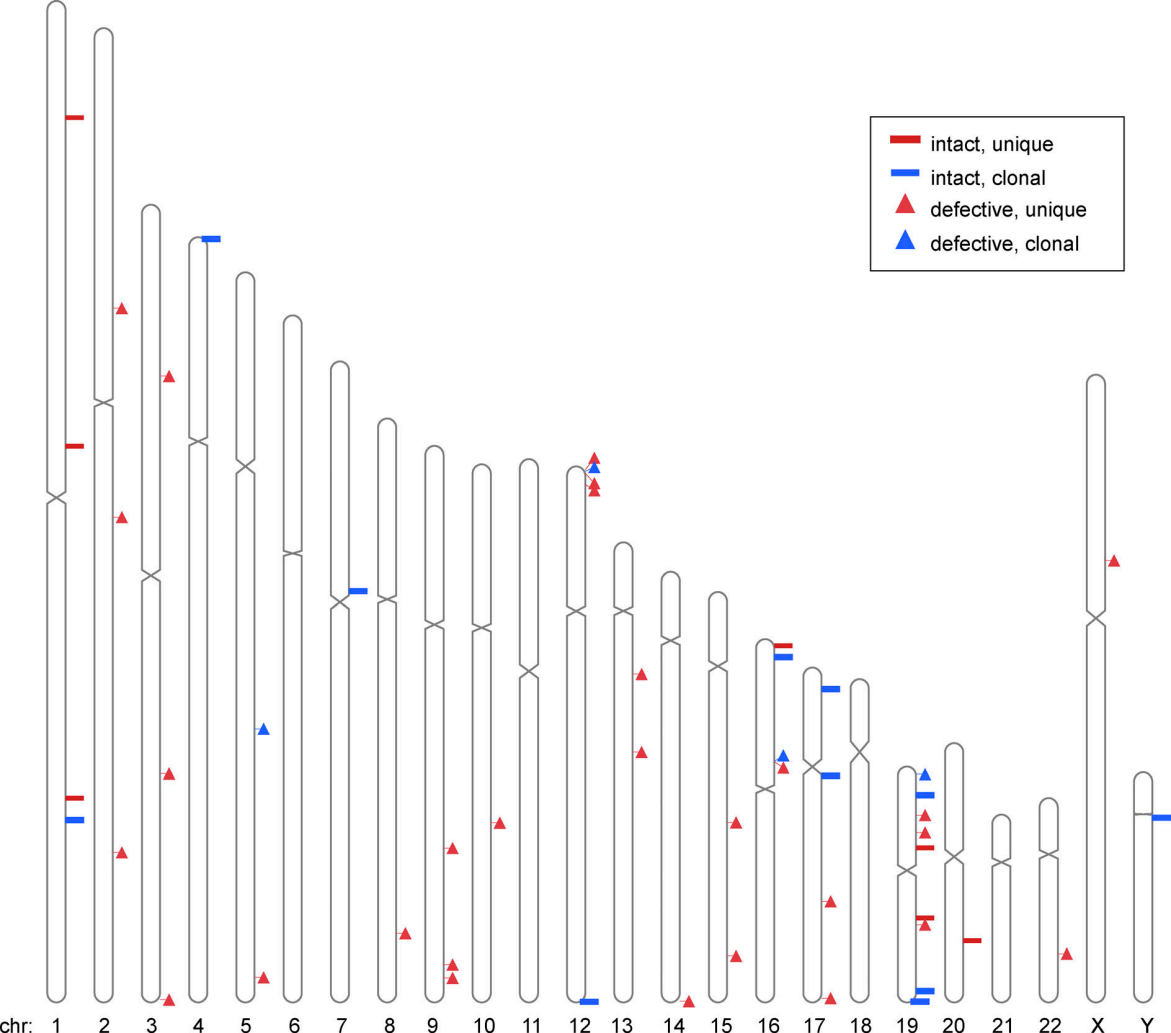
**Ideogram of positions of mapped integrations.** Diagrammatic representation of chromosomal locations of intact and defective; clonal and unique proviruses. Chr, chromosome.

In agreement with studies of global retroviral DNA integration, HIV-1 integration was enriched on chromosome 19 (Fig. 3 A and Fig. S1; Schröder et al., 2002; Vansant et al., 2020). Integrations were more frequent in genic regions, but there was no significant difference (P = 0.38; two-tailed Fisher’s exact test) between defective and intact integrations. In addition, we found no difference between intact and defective proviruses with respect to their position in introns and exons (P = 0.495; two-tailed Fisher’s exact test; Fig. 3 B). Furthermore, intact and defective proviruses were in the same relative orientation to the proximal transcriptional start site (TSS; Fig. 3 C) and at similar distances from the TSS (Fig. 3 D). Both defective and intact proviruses favored integration in the proximity of repetitive short interspersed nuclear elements (Fig. 3 E), with no difference in relative distance to the proximal repetitive element (Fig. 3 F). Whereas we found only one intact and no defective proviruses in centromeric satellite region DNA, this site of integration is characteristic of intact proviruses in ECs (Fig. 3 E; Jiang et al., 2020).

**Figure S2.**
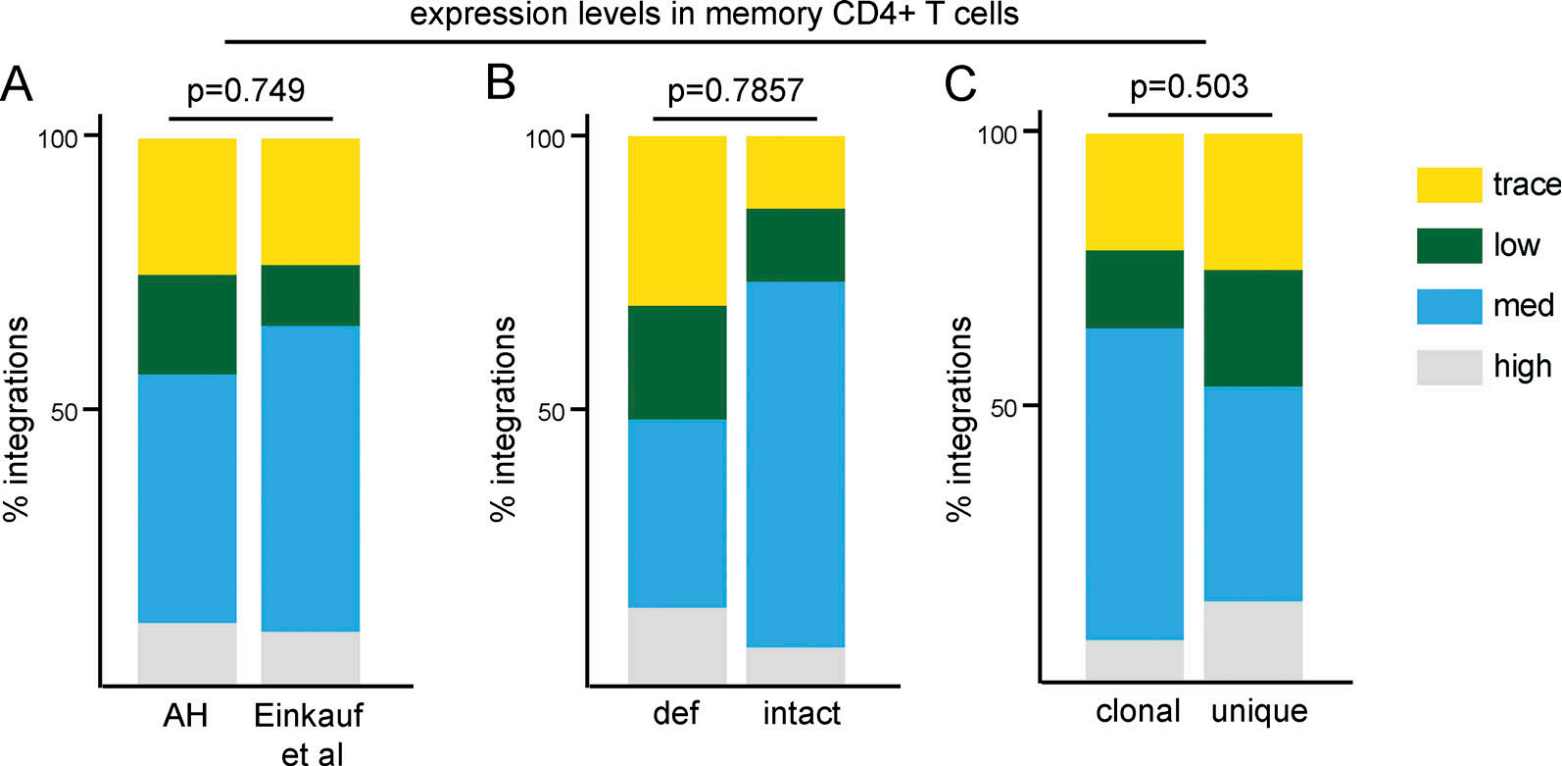
**Gene expression in resting in memory CD4^+^ T cells in the vicinity of integration. (A)** Proportion of all proviral integrations in genes with low, medium (med), or high expression. Cut-offs determined by upper quartile, median, lower quartile, and minimum expression values. Comparison of integrations in this study (AH) and previous publications (Einkauf et al., 2019). **(B)** Proportion of defective (def) and intact proviral integrations mapped to genes with trace, low, medium, or high expression (see Table S2). **(C)** Proportion of clonal and nonclonal proviral integrations mapped to genes with trace, low, medium, or high expression (see Table S2). All P values refer to proportion of integrations in highly expressed genes, determined by two-proportion Z-test.

We used publicly available data on memory CD4^+^ T cells to assess gene expression and epigenetic features in the vicinity of mapped integrations because the latent reservoir resides primarily in this T cell subset (Fromentin and Chomont, 2021; Murray et al., 2016). Across all categories, integrations were predominantly mapped within genes that are moderately or highly expressed in memory CD4^+^ T cells (Fig. S2). While there is no difference in gene expression levels between integrations of intact versus defective proviruses, three-dimensional chromosomal interaction data from chromatin conformation capture (Hi-C) sequencing shows intact proviruses are less frequently found in the active chromatin compartments compared with their defective counterparts (Fig. 3 G; Rao et al., 2014). Despite the transcriptional activity, the sites of both intact and defective integrations were associated with high levels of methylation that typically indicate epigenetic repression (Fig. 3 H; Komaki et al., 2018).

**Figure 4.**
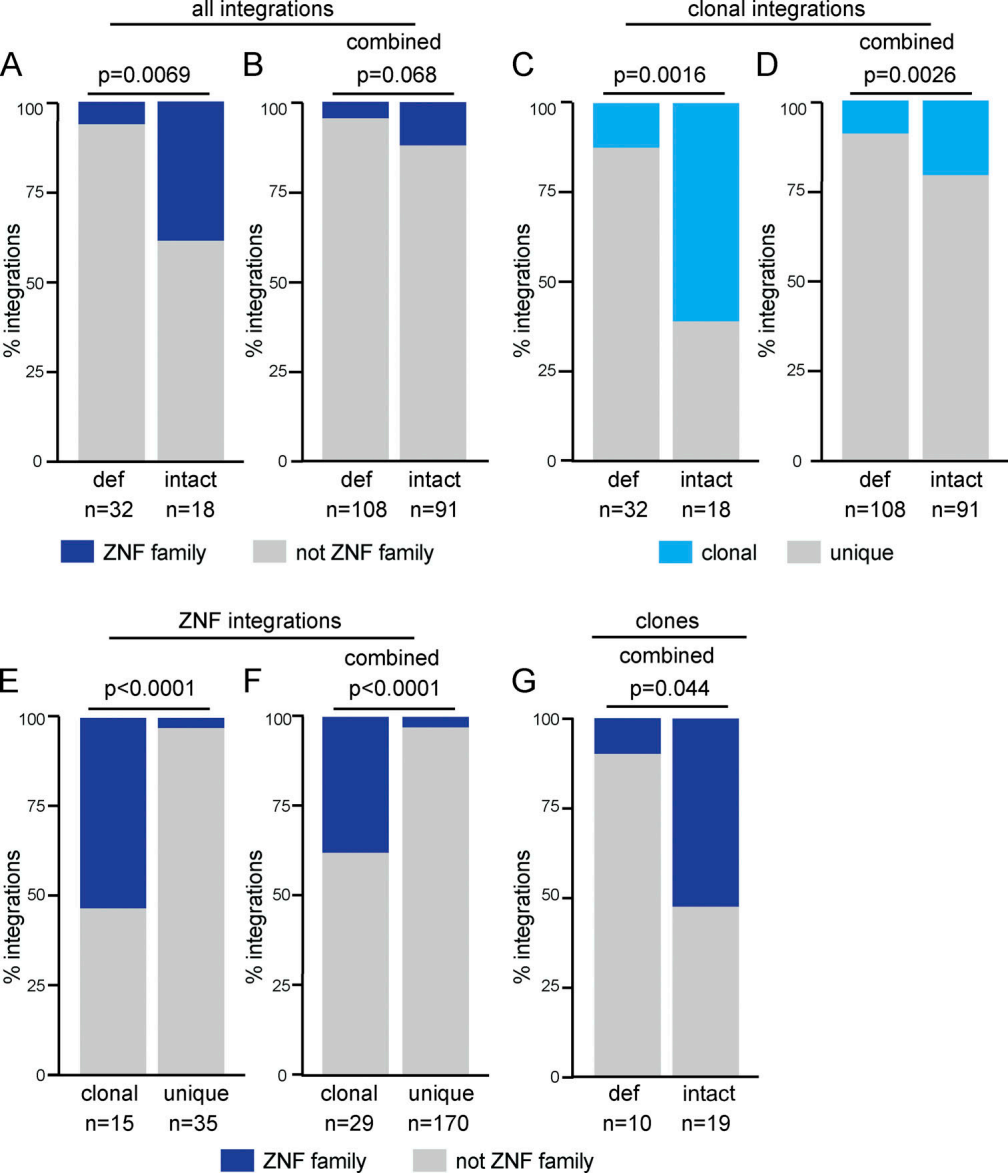
**Frequency of integrations into ZNF genes. (A and B)** Proportion of intact and defective (def) proviral integrations mapped to ZNF genes in long-term ART-treated individuals mapped (A) in this study and (B) in combination with long-term ART-treated individuals reported by others (Einkauf et al., 2019). **(C and D)** Proportion of clonal versus nonclonal defective and intact integrations in long-term ART-treated individuals mapped (C) in this study and (D) in combination with long-term ART-treated individuals reported by others (Einkauf et al., 2019).** (E and F) **Proportion of clonal and nonclonal integrations mapped in ZNF genes found in long-term ART-treated individuals (E) in this study and (F) in combination with long-term ART-treated individuals reported by others (Einkauf et al., 2019). **(G)** Proportion of defective and intact clones in ZNF genes from combined analysis with long-term ART-treated individuals reported by others (Einkauf et al., 2019). All P values determined by two-tailed Fisher’s exact test.

When examined individually, there was a notable preponderance of intact integrations into ZNF family genes, particularly among expanded clones (Fig. 2). 61% of intact and 13% of defective proviral integrations were found in expanded clones (Fig. 2 and Fig. 4 C). Among these clonal proviral integrations, 39% and 6% of the intact and defective proviruses, respectively, were integrated into ZNF genes (Fig. 2 and Fig. 4 E). This finding is best illustrated in participant B207, whose latent reservoir is largely populated by clones of various sizes. All four intact proviral clones in this individual were inducible in vitro, and three of the four integrated proviruses mapped to genes in the ZNF family (Fig. 2). Moreover, we note that in study participants whose reservoirs contain proviral clones mapped within ZNF genes, the specific clones with integrations in ZNF genes appear to be the most expanded (Fig. 2).

To better understand the apparent association between intact clonally expanded proviruses and ZNF genes, we combined our dataset with other published data obtained from long-term ART-suppressed HIV-infected individuals (Einkauf et al., 2019). Our intact proviral integrations are significantly enriched in ZNF genes (P = 0.0069; Fig. 4 A). However, when all the available data are combined, this enrichment is less pronounced (P = 0.068; Fig. 4 B). To understand this potential discrepancy, we analyzed clonality in the two datasets. In contrast to our dataset, in which there is a preponderance of clonal integrations (61% of intact and 13% of defective integrations), only a minority of the 199 unique integrations in the combined dataset (18.8% of intact and 9.4% of defective integrations) were clonal (Fig. 4 D). We found the proportion of ZNF integration within clones is significantly enriched (P < 0.001) compared with unique integrations (Fig. 4, E and F). When all intact clonal integrations are combined, 11 of 29 were in ZNF genes, which is a significant enrichment when compared with defective clonal integrations (P = 0.044; Fig. 4 G). Thus, intact proviruses found in expanded clones of CD4^+^ T cells are preferentially integrated in distinct sites in the host genome.

**Figure 5.**
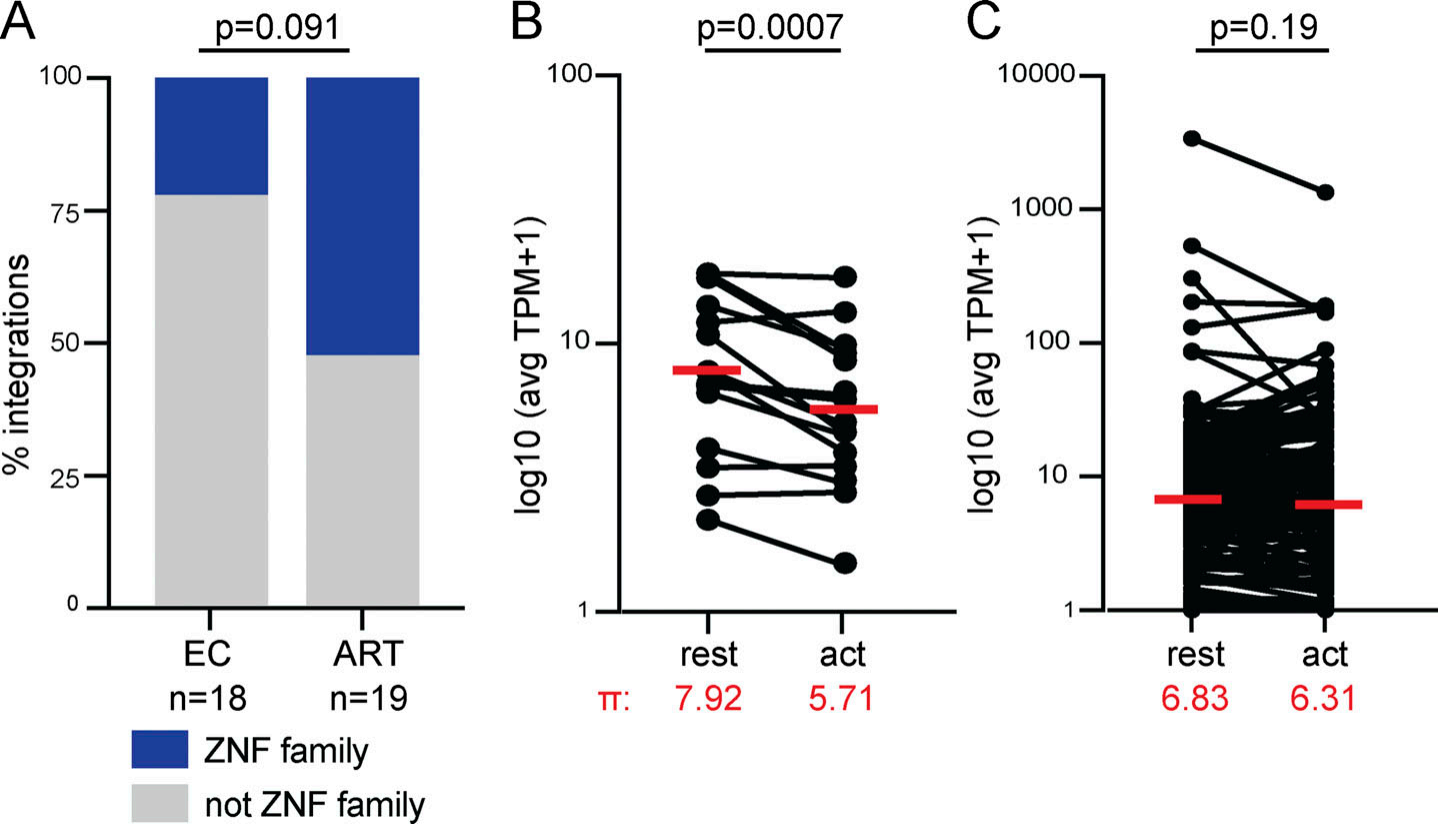
**Transcriptional activity of ZNF genes. (A)** Comparison of proviral integration sites in intact proviral clones from ECs and ART-treated individuals (Einkauf et al., 2019; Jiang et al., 2020). Fraction of clones obtained from chronically infected ART-treated individuals and EC integrated in ZNF genes represented in blue. Two-tailed Fisher’s exact test. **(B)** Combined analysis of mapped integrations from this study and previously published integration data from ECs and individuals on long-term ART (Einkauf et al., 2019; Jiang et al., 2020). Comparison of normalized expression at rest and upon activation (act) in primary memory CD4^+^ T cells for ZNF genes with mapped integrations. Geometric mean (π) of expression levels noted in red (Cano-Gamez et al., 2020). **(C)** Comparison of normalized expression at rest and upon activation (act) in primary memory CD4^+^ T cells for non-ZNF genes with mapped integrations (Cano-Gamez et al., 2020). Geometric mean (π) of expression levels noted in red. P values determined by Wilcoxon signed-rank test. avg, average; TPM, transcripts per million.

Preferential integration into ZNF family genes on chromosome 19 is associated with viral reservoirs in HIV-1 ECs that durably control viral replication without therapy (Jiang et al., 2020). The reservoir in these individuals is also enriched in large expanded clones. To determine whether this is specific to the integration landscape of ECs or can be generalized to all integrations in expanded clones of CD4^+^ T cells, we compared the relative proportion of intact clonal proviruses found in ZNF genes in ART-treated individuals and ECs. There was no significant difference in the proportion of ZNF integrations between the two groups, suggesting that this is a feature of expanded clones of proviruses and not limited to ECs (Fig. 5 A).

**Figure S3.**
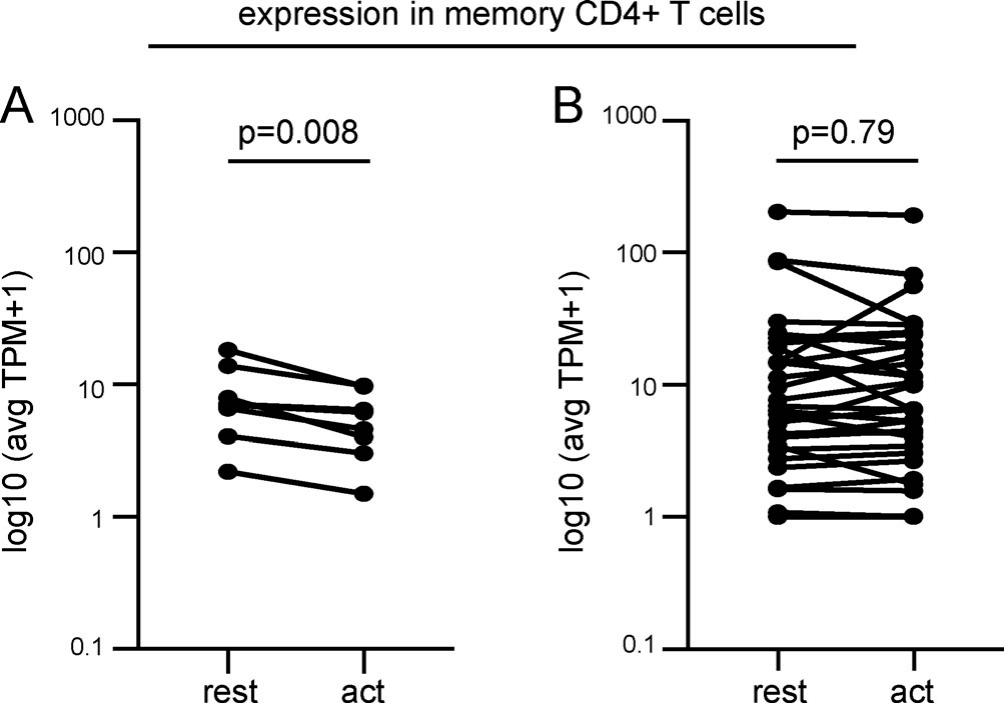
**Comparison of expression levels in ZNF and non-ZNF genes in memory CD4^+^ T cells with integrations in this study alone.** **(A)** Expression of ZNF genes with mapped integrations, at rest and upon activation with α-CD3/α-CD28 human T-Activator Dynabeads (act). **(B)** Expression of non-ZNF genes with mapped integrations, at rest and upon activation (act; see Table S2). P values determined by Wilcoxon signed-rank test. avg, average; TPM, transcripts per million.

ZNF genes are associated with repressive chromatin marks in CD4^+^ memory T cells. Nevertheless, ZNF genes are expressed in these cells (Fig. S2). To determine whether ZNF genes associated with clonal integrations might be remodeled to repress transcription upon cellular activation, we examined RNA sequencing data comparing resting and activated memory CD4^+^ T cells (Cano-Gamez et al., 2020). ZNF genes that carry intact integrations are repressed upon memory T cell activation (P = 0.0007; Fig. 5 B and Fig. S3). In contrast, the transcriptional activity of non-ZNF genes that carry proviral integrations is unaltered by memory CD4^+^ T cell activation (Fig. 5 C and Fig. S3). In summary, the data suggest that integration into ZNF genes provides a survival advantage that favors clonal expansion.

## Discussion

Understanding the mechanisms that mediate HIV-1 latency is critical to developing curative strategies. Recent advances in sequencing technologies have demonstrated that the latent reservoir is dynamic, and it is maintained by proliferation of latent HIV-1–infected cells (Cohn et al., 2020; Cohn et al., 2015; Gantner et al., 2020; Hosmane et al., 2017; Liu et al., 2020a; Lorenzi et al., 2016; Simonetti et al., 2021). Indeed, the majority of intact replication competent latent HIV-1 proviruses are found in expanded clones of CD4^+^ T cells in chronically infected individuals who are suppressed on ART (Hosmane et al., 2017; Lorenzi et al., 2016). Changes in the quality of the reservoir over time reflect fluctuations in the size of CD4^+^ T cell clones infected with genetically identical HIV-1 proviruses (Cohn et al., 2018; Simonetti et al., 2021). This study provides evidence that integration of latent HIV-1 proviruses is significantly enriched in ZNF genes in clonally expanded CD4^+^ T cells.

Expression of latent HIV-1 proviruses can be induced in CD4^+^ T cells by stimuli that promote cell division (Laird et al., 2013; Siliciano and Siliciano, 2005). However, the latent reservoir evolves over time in individuals on suppressive ART such that an initially diverse collection of latent HIV-1 proviruses tends to become more and more clonal with time (Antar et al., 2020; Cohn et al., 2015; Hosmane et al., 2017; Lorenzi et al., 2016; Pinzone et al., 2019; Reeves et al., 2018). Reservoir size remains relatively stable, in part because proliferation and clonal expansion of infected memory CD4^+^ T cells in response to chronic antigen exposure is offset by cell death (Liu et al., 2020a; Mendoza et al., 2020; Simonetti et al., 2021). Given the dynamic nature of the reservoir, the surviving latent proviruses must be selected for resistance to reactivation by signals that induce CD4^+^ T cell division. Consistent with this idea, the probability of latent HIV-1 reactivation is inversely related to the size of the expanded CD4^+^ T cell population (Lorenzi et al., 2016; Wang et al., 2018).

Given the cytopathic effects of HIV-1 gene expression, expansion of latent cells must be accompanied by persistent silencing of viral transcription. Whether silencing is mediated by repressors of HIV-1 transcription, the genomic context of HIV-1 integration, or both remains to be determined (Cohn et al., 2018; Einkauf et al., 2019; Lewinski et al., 2005; Lewinski et al., 2006). To understand how integration into the genome might contribute to survival of clonal latent cells, we studied chronically infected participants with large intact reservoirs dominated by expanded clones of latent HIV-1 proviruses. Overall, the genomic characteristics of our intact integrations are consistent with previous studies (Anderson and Maldarelli, 2018). Integrations are enriched in genic regions, and in chromosomes 1 and 19, with no differences between intact and defective proviruses in terms of introns and exons, relative orientation, or distance to TSSs (Anderson and Maldarelli, 2018; Schröder et al., 2002).

**Figure S4.**
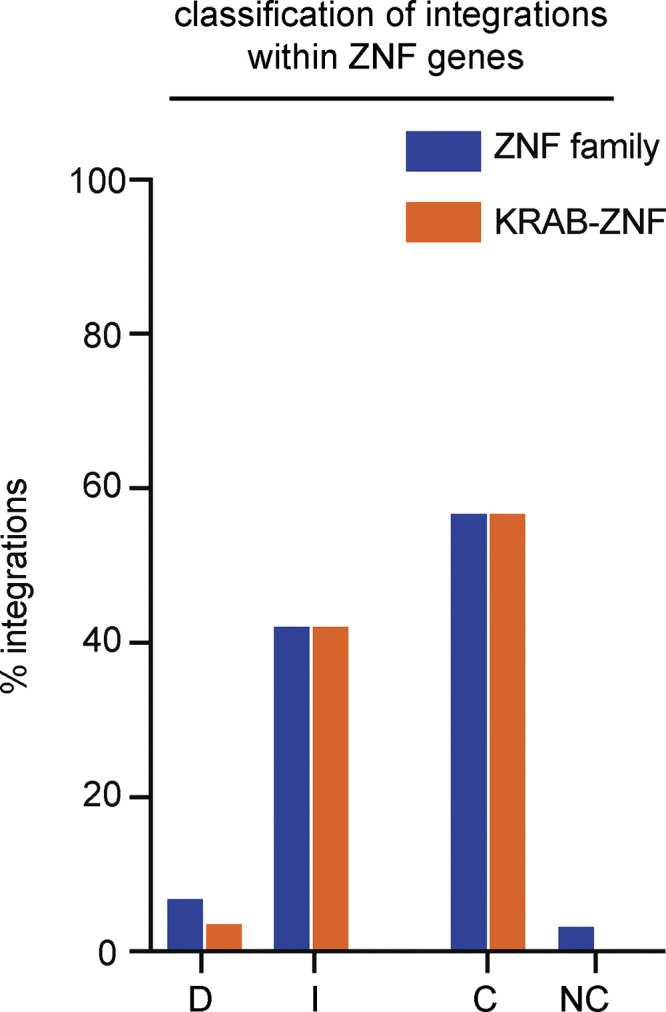
**Sub-classification ZNF integrations.** Proportion of defective (D), intact (I), clonal (C), or nonclonal (NC) ZNF integrations found in KRAB-ZNF genes.

In contrast to other HIV-1–infected individuals, intact proviral integrations were shown to favor KRAB-ZNF genes in ECs, suggesting that this type of integration might contribute to control of HIV-1 infection (Jiang et al., 2020). Our data indicate that this is a more general feature of integrated intact proviruses in expanded clones of CD4^+^ T cells. In contrast, intact nonclonal and defective proviral integrations are not enriched in the KRAB-ZNF family of transcriptional repressors (Fig. S4). Defective provirus integrations are enriched in proximity to some cancer-associated genes such as *BACH2*, *MKL2*, and *STAT5B*, but whether integration in the proximity of such genes favors clonal expansion remains to be determined (Liu et al., 2020b; Maldarelli et al., 2014; Wagner et al., 2014).

HIV-1 integration favors actively transcribed regions of the genome (Craigie and Bushman, 2012; Schröder et al., 2002). Consistent with this idea, ZNF genes are actively transcribed in resting memory CD4^+^ T cells (Cano-Gamez et al., 2020; Einkauf et al., 2019; Jiang et al., 2020). Although additional mechanistic studies are necessary to show how ZNF gene expression might impact proviral transcription, we hypothesize that silencing KRAB-ZNF family locus transcription during cellular activation favors latency, thereby preventing HIV-1–mediated cytopathic effects and enabling clonal expansion. Conversely, proviral integrations in genes that are transcriptionally activated during CD4^+^ T cell stimulation might be eliminated over time (Chen et al., 2017).

The extent to which expanded clones with proviral integrations contribute to viral rebound following treatment interruption remains unclear. Rebound viruses are typically distinct from clonally expanded and outgrowth viruses, possibly due to suppression by autologous neutralizing antibodies (Bertagnolli et al., 2020) and CD8^+^ T cell responses (Bar et al., 2016; Cohen et al., 2018; Lu et al., 2018; Mendoza et al., 2018). In conclusion, the data indicate that the site of proviral integration provides a selective advantage for clonal expansion and survival of latently infected CD4^+^ T cells.

## Materials and methods

### Study participants

All study participants were recruited by the Rockefeller University Hospital, New York, NY, and the University Hospital Cologne, Cologne, Germany. Individuals 9254 and 9255 were participants in analytical treatment interruption (ATI) trial NCT02825797. Individuals 5203 and 5104 are participants in ATI trial NCT03526848. Individuals 603 and B207 did not undergo ATI. Informed consent was obtained from all subjects. All relevant ethical regulations were followed. Peripheral blood mononuclear cells (PBMCs) were isolated from leukapheresis performed according to protocols approved at The Rockefeller University by the Rockefeller Internal Review Board.

### Quantitative and qualitative viral outgrowth assay

Viral outgrowth was performed as previously described (Lorenzi et al., 2016).

### Whole genome amplification

Genomic DNA was purified from CD4^+^ T cells isolated from bulk PBMCs (Miltenyi Biotec; 130–096-533) by phenol-chloroform extraction and ethanol precipitation. To quantify total HIV-1 proviral frequency, genomic DNA (gDNA) was assayed for *gag* by quantitative PCR (Gaebler et al., 2019). gDNA was diluted to single viral genome levels based on *gag* quantitative PCR results, such that <30% of wells were *gag*^+^. gDNA was subjected to multiple displacement amplification (MDA) with ϕ29 polymerase at 30°C for 4 h (Qiagen; 150345). Subsequently, MDA products in each well were diluted 1:10 with nuclease-free water and subject to viral sequencing. Different plates were processed independently, and we did not observe differences between plates within each individual sampled.

### HIV-1 NFL sequencing

NFL HIV-1 sequences were amplified from 1.5 μl of diluted MDA products using a nested two-amplicon (Ho et al., 2013) or a nested five-amplicon method (Einkauf et al., 2019). PCR amplicons were subject to paired-end sequencing using Illumina MiSeq Nano 300 V2 cycle kits at a concentration of 12 pM. HIV-1 sequences were reconstructed and classified as intact or defective using our in-house pipeline (Defective and Intact HIV Genome Assembler), as previously described (Mendoza et al., 2020). Clones were defined by aligning sequences classified as intact, MSD mutation, non-functional, or missing internal genes to HXB2 calculating the hamming distance. Sequences containing three or fewer differences between the first nucleotide of *gag* and the last nucleotide of *nef* were classified as members of the same clone.

### Integration library construction

To obtain sufficient material for integration library construction, a second-round whole genome amplification was performed from initial MDA products. To enrich for amplicons containing the 3′ HIV-1 and human sequence, 1 μl of 50 µM 15UTRi-F 5′-CTA​GGG​AAC​CCA​CTG-3′ was spiked into the MDA reaction. Second-round MDA products were purified using AMPure XP beads (Beckman Coulter; A63880), and ∼10 µg of DNA was sonicated to 300–1,500 bp. Fragments were ligated to 200 pM of annealed linker, digested with BglII (NEB; R0144) to remove HIV-1 sequences, and bead-purified. For each sample, DNA was divided into 300-ng aliquots and amplified by linear PCR with biotinylated LTR1 5′-CTT​AAG​CCT​CAA​TAA​AGC​TTG​CCT​TGA​G-3′ (1 × [98°C, 1 min] 17 × [98°C, 15 s; 62°C, 30 s; 72°C, 30 s] 1 × [72°C, 5 min]). Reactions were spiked with p115 5′-GCA​GCG​GAT​AAC​AAT​TTC​ACA​CAG​GAC-3′ and further amplified as follows: 1 × (98°C, 1 min) 25 × (98°C, 15 s; 62°C, 30 s; 72°C, 30 s) 1 × (72°C, 5 min). Products >300 bp were purified using AMPure XP and enriched by magnetic streptavidin beads. DNA was amplified off the magnetic beads by semi-nested PCR using LTR2 (same cycling conditions as above) before a spike-in of p115 and additional cycles. PCR products were purified with AMPureXP, blunted (Lucigen; ER81050), and ligated with NextFlex paired-end adapters diluted 1:10 (PerkinElmer; NOVA-514102). Adapter-ligated fragments were PCR-enriched with NextFlex primers: 1 × (98°C, 1 min) 27× (98°C, 15 s; 66°C, 30 s; 72°C, 30 s) 1 × (72°C, 5 min). Amplicons from 300–1,500 bp were gel-purified and sequenced by 150 bp paired-end sequencing on Illumina MiSeq or Illumina NextSeq 500. All PCR reactions were performed with High Fidelity Phusion polymerase (Thermo Fisher Scientific; F530L)

### Computational pipeline for mapping HIV integration sites

Reads starting with the last 34 nucleotides of the HIV LTR (bait reads) and corresponding pairs starting with the first 43 nucleotides of the p115 linker (target reads) were extracted from raw sequencing file using cutadapt v2.10 (https://github.com/marcelm/cutadapt/) and seqtk (https://github.com/lh3/seqtk).

Bait reads were mapped to the HIV LTR using Smalt (https://www.sanger.ac.uk/tool/smalt-0/), and only read pairs containing identical 34 nucleotides were selected for downstream processing.

The paired-end reads were mapped to the human genome assembly GRCh38 after trimming both LTR and the p115 linker sequences by BBduk from BBTools (https://jgi.doe.gov/data-and-tools/bbtools/). Once mapped, reads were subjected to a two-step filtering process using in-house Perl scripts. The first step, a distance-based filter, selects the most reliable PCR fragments generated by the 3′LTR and p115 primers. It consists of selecting reads overlapping a maximum of 15 nucleotides or mapped at a maximum distance of 2 kb from each other. The second step, an alignment-based filter, determines reads resembling bona fide HIV-1 integrations in the human genome. Paired-end reads are selected if the bait read met the following criteria: (1) at least 30 perfect matches with the human genome, (2) soft-clipping between 1 and 3 nucleotides, at least 27 identical nucleotides in the remaining sequence, and finally (3) 1 match in the first nucleotide followed by 1 or 2 mismatches and >28 matches in the remaining sequence. Reads mapping in the same position and orientation were combined into a single putative PCR fragment amplified by the LTR and p115 primers. The number of reads collapsed into one fragment reflects the score of the respective fragment. Fragments supported by a single alignment were not considered in the analysis. At the end of the pipeline execution, integration hotspots were calculated based on a definition of hotspots established by previous work on translocation-capture sequencing by our group (Klein et al., 2011).

Parallel execution and computing resource management were performed by the Snakemake framework (Köster and Rahmann, 2018).

### Combined analysis with published integration datasets

The published dataset for integration sites during long-term ART was generated from PBMCs of three virally suppressed HIV-infected individuals recruited at Massachusetts General Hospital (Boston, MA), Brigham and Women’s Hospital (Boston, MA), and the National Institutes of Health Clinical Center (Bethesda, MD), and who have been on continuous ART for 8–13 yr at the time of PBMC sampling (Einkauf et al., 2019). Integration sites were mapped using integration site loop amplification, nonrestrictive linear amplification-mediated PCR, or ligation-mediated PCR (Lenti-X Integration Site Analysis Kit; Clontech; cat. 631263; Paruzynski et al., 2010; Wagner et al., 2014). The published dataset for integration sites in ECs was generated from PBMCs of 11 HIV-infected ECs recruited at Massachusetts General Hospital, Brigham and Women’s Hospital, and at the University of California, San Francisco, at the Zuckerberg San Francisco General Hospital (Jiang et al., 2020). In both studies, DNA from enriched CD4^+^ T cells was amplified and quantified using digital droplet PCR (BioRad) to detect a 127-bp 5′LTR-*gag* amplicon (Lee et al., 2017).

### Computational analysis of HIV integration sites

HIV integration sites were annotated by in-house Perl scripts using GENCODE v32 and RepeatMasker tracks downloaded from the University of California, Santa Cruz Genome Browser (https://genome.ucsc.edu/cgi-bin/hgTables). Because more than one transcript may be equally distant from the integration site, a priority level was defined to select one transcript representing a possible affected gene. We selected transcripts if the HIV integration was (1) inside an exon, (2) inside an intron, or (3) in a nongenic region. For overlapping transcripts, we selected the one containing the closest TSS.

We obtained information on gene expression in different T cell subtypes from the Database of Immune Cell Expression, Expression of quantitative trait loci and Epigenomics (Schmiedel et al., 2018) and from bulk RNA sequencing data generated by Cano-Gamez et al. (2020). Genes were divided into silent (0 transcripts per million) and expressed genes. We observed a bimodal distribution on the expressed genes. Expressed genes were subdivided into trace-expressed genes and more highly expressed genes using a normal mixture model implemented by the mclust R package (Scrucca et al., 2016). The higher group was further divided into tertiles, representing low, medium, and highly expressed genes.

Epigenetics analyses were performed by integrating data from three different sources: (1) methylation from the iMethyl database (Komaki et al., 2018), (2) chromatin immunoprecipitation sequencing data for H3K4me1 and H3K9me3 histone marks from the Roadmap Epigenomics Consortium repository (Kundaje et al., 2015), and Hi-C data generated by Rao et al. (2014).

### Online supplemental material

Fig. S1 shows chromosomal positions of all mapped integrations in this study. Fig. S2 shows the expression levels in memory CD4^+^ T cells of genes with mapped integrations. Fig. S3 shows expression levels in resting and activated memory CD4^+^ T cells of genes with mapped integrations. Fig. S4 shows proportion of integrations within ZNF and KRAB-ZNF genes. Table S1 shows demographics and clinical features of study participants. Table S2 lists positions and genomic characteristics of all integrations mapped in this study.

## Supplementary Material

Table S1 shows demographics and clinical features of study participants.

Table S2 lists positions and genomic characteristics of all integrations mapped in this study.
